# 

**Published:** 1880-11

**Authors:** 


Bound Volume of 1880.—Upon the completion of the current volume of *The Independent Practitioner*, we will have a limited number of the volume bound in cloth, which can be had for $2.75. As an inducement to those who have not received it this year, and wish to subscribe for Vol. II, 1881, we will mail the bound volume to them and send the issues of volume II regularly, for $4.25. B. M. W.

### HYMENEAL.

"Domestic happiness, thou only bliss
Of paradise that has survived the fall."

TURNER—DOUGHTY.—On October 14th, by Dr. John Leyburn, at the Associate Reformed Church, Dr. W. D. Turner, of Va., to Sallie A., daughter of T. P. Doughty, Esq., of this city.

---

### NECROLOGICAL.

"Death is the crown of life:
Were death denied, poor man would live in vain."

On the evening of the 26th of October, 1880, Dr. James W. Rowland breathed his last at the Union Protestant Infirmary in this city, in the 84th year of his age.

Many persons who receive pamphlets, papers, etc., through the mails are unaware of the United States laws relating to them, and such will feel indebted to us for the information and guidance the enactments will afford which we copy below, viz:

1. A postmaster is required to give notice *by letter* (returning a paper does not answer the law) when a subscriber does not take his paper out of the office, and state the reason for its not being taken. Any neglect to do so makes the postmaster *responsible* to the publishers for payment.

2. Any person who takes a paper from the post-office, whether directed to his name or another, or whether he has subscribed or not, is responsible for the pay.

3. If a person orders his paper discontinued, he must pay all arrearges, or the publisher may continue to send it until payment is made, and collect the whole amount, *whether it be taken from the office or not.* There can be no legal discontinuance until the payment is made.

4. If the subscriber orders his paper to be stopped at a certain time, and the publisher continue to send, the subscriber is bound to pay for it *if he takes it out of the post-office.* The law proceeds upon the ground that a man must pay for what he uses.

5. The courts have decided that refusing to take a newspaper and periodicals from the post-office or removing and leaving them uncalled for, is *prima facie* evidence of intentional fraud.

---

This column will be sold for advertising space six month during the year at *special rates.*

N. B.—Each month THE INDEPENDENT PRACTITIONER will be received by a large number of persons for the *first* time, all of whom we trust will be induced to become subscribers. They will please remit NOW the amount of subscription, ($2.00) to insure the continuation of the journal.

